# A review of supportive care for older people with advanced chronic kidney disease

**DOI:** 10.1093/ckj/sfac256

**Published:** 2022-12-07

**Authors:** Ted J FitzGerald, Hanneke Joosten, Marjolijn van Buren, Katie Vinen, Edwina A Brown

**Affiliations:** Department of Nephrology, Imperial College Hospital's NHS Trust, London, UK; Department of Nephrology, Maastricht UMC, Maastricht, The Netherlands; Department of Nephrology, Leiden UMC, Leiden, The Netherlands; Department of Nephrology, King's College Hospital NHS Trust, London, UK; Department of Nephrology, Imperial College Hospital's NHS Trust, London, UK

**Keywords:** age, CKD, elderly, ESRD, prognosis

## Abstract

Supportive care (SC) is a multidimensional and person-centred approach to managing advanced CKD that engages the person and their caregivers in shared decision making from the outset. Rather than focusing on disease-specific therapies, SC is a collection of adjuvant interventions and adaptations to conventional treatments that can be used to improve the individual's quality of life. Recognizing that frailty, multi-morbidity and polypharmacy are more common among older people with advanced chronic kidney disease (CKD) and that people in this group tend to prioritize quality of life over survival as a goal of care, SC represents an important adjunct to disease-specific therapies in CKD management. This review provides an overview of SC in the older person with advanced CKD.

## INTRODUCTION

Frailty, multi-morbidity and polypharmacy are more common among older people with advanced chronic kidney disease (CKD) [1–3], and contribute to complexity in decision-making as the disease progresses. Current evidence suggests that health-related quality of life (HRQoL) and symptom burden are similar for dialysis and non-dialysis approaches to end-stage kidney disease (ESKD) management in older adults. Although dialysis may improve survival compared with conservative management, this benefit is largely mitigated amongst the oldest patients and those living with significant comorbidity [4–7]. Furthermore, older adults tend to experience functional and cognitive decline after dialysis initiation, as well as an increased risk for hospitalization [5, 8, 9]. Considering these factors and acknowledging that older adults with ESKD are more likely to prioritize HRQoL outcomes over survival [10], treatment strategies should emphasize HRQoL as a priority.

This review will focus on SC strategies in the older adult with advanced CKD [estimated glomerular filtration rate (eGFR) is ≤20 mL/min/1.73 m^2^]. It provides an overview of shared-decision making (SDM), advance care planning (ACP), conservative kidney management (CKM) and supportive dialysis regimens. Finally, it describes a framework for the clinical training and infrastructure required to provide SC in nephrology practice.

## WHAT IS SUPPORTIVE KIDNEY CARE?

SC is a multidimensional and person-centred approach to managing advanced CKD that engages the person and their caregivers in shared decision making from the outset. Rather than focusing purely on disease-specific therapies (i.e. dialysis, transplantation, immunosuppression), SC is a collection of adjuvant interventions and adaptations to conventional treatments that can be used to improve the individual's quality of life.

**Figure 1:**
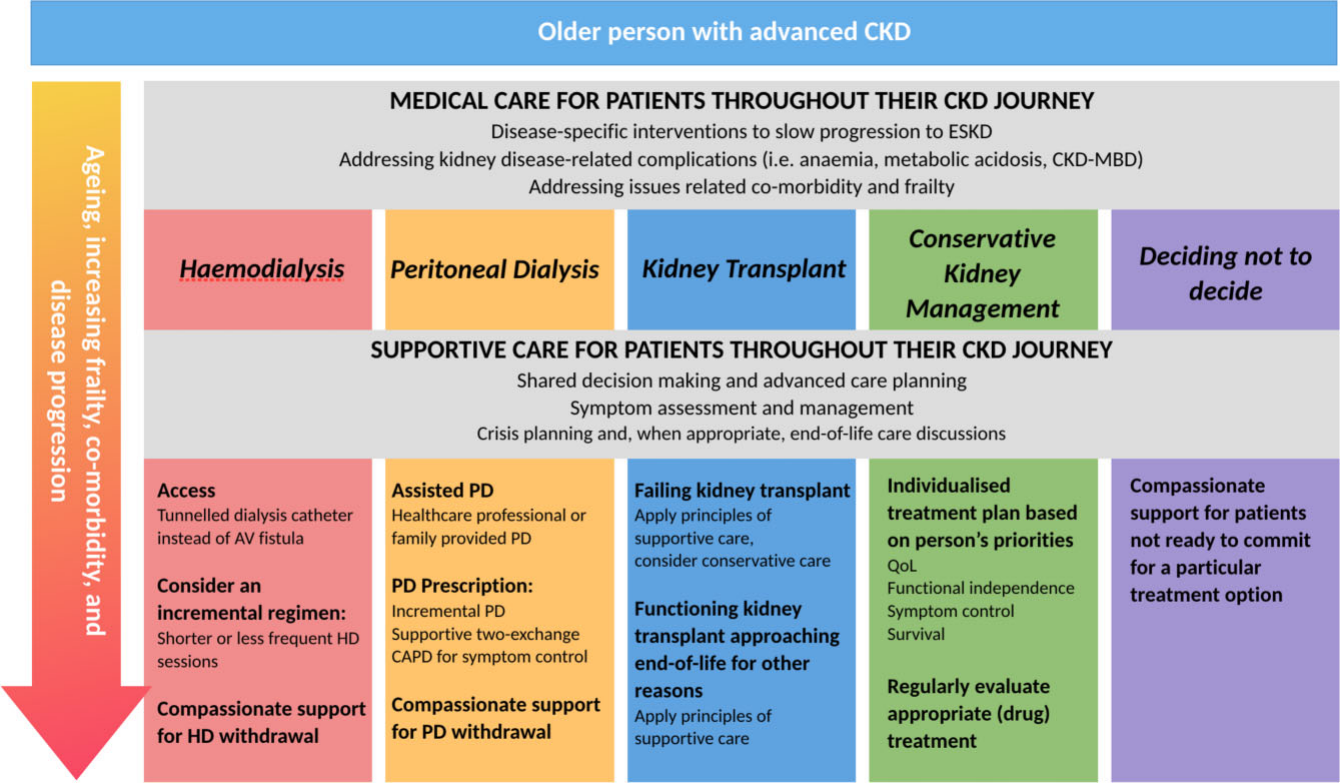
SC is central to the management of all older people with progressive and advanced CKD, whose survival and HRQoL is negatively impacted by their diagnosis.

In this article, we propose that SC strategies are an important adjunct in the management of ESKD in older adults, regardless of disease-specific treatment (see Fig. 1), and that the term should not be considered synonymous with conservative or non-dialysis therapy (which represents a specific treatment decision in ESKD).

## SYMPTOM ASSESSMENT AND MANAGEMENT

The symptom experience of the older person with ESKD is a composite of uraemia, fluid overload, comorbidity, frailty, polypharmacy and psychosocial issues [11, 12]. This likely explains why dialysis does not ameliorate many of the unpleasant symptoms associated with advanced CKD, and why symptom management strategies are an important adjunct to ESKD care in older people [11, 13]. Table 1 provides a brief overview of the common symptoms in advanced CKD and some management strategies that might be considered.

**Table 1:** Symptom management in advanced CKD

Symptom	Non-pharmacological measures	Pharmacological management	Other	Caveats
Pruritus	• Topical agents (emollients, capsaicin)	• Low-dose gabapentinoids	• UVB therapy	
		• Difelikafelin		
Sleep disorders	• Promote better sleep hygiene	• Simple sedatives		• Try to avoid sedatives in older adults where possible
	• Managing concurrent symptoms (e.g. restless legs, depression)			
RLS	• Avoid excessive ultrafiltration during dialysis sessions	• Trial low-dose dopamine agonists (e.g. mirapexin)	• Address hyperphosphataemia and iron deficiency	• Assess efficacy of any pharmacological intervention after a suitable interval
		• Low-dose gabapentinoids		
Depression	• Cognitive behavioural therapy	• Oral antidepressants		
Pain	• Determine likely aetiology and assess severity	• Use the WHO Analgesia Ladder (adjusted for CKD) for a stepwise approach to pain management	• Consider co-existing depression	• Try to limit use of opioids in older adults• Try to avoid tramadol
		• In cases of neuropathic pain, consider low-dose gabapentinoids which may also treat other symptoms associated with ESKD		

RLS: restless legs syndrome; WHO: World Health Organization.

A stepwise approach to symptom management has been proposed, whereby non-pharmacological interventions are used first-line to reduce polypharmacy. Pharmacological therapies are used second-line if conservative measures do not achieve adequate symptom control. These should be introduced at a low dose, with interval assessment of their efficacy, and should ideally target several symptoms. For example, low-dose gabapentin has been shown to demonstrate efficacy in the treatment of uraemic pruritus and restless legs syndrome [11, 14]. Davison *et al*. provide an excellent evidence-based synthesis of current symptom management approaches in CKD [14].

Person-reported outcome measures (PROMs) permit a standardized approach to symptom identification and may improve symptom recognition among patients and clinicians [15]. Currently, there is limited evidence to support the preferential use of a particular PROM for symptom screening in older adults with ESKD [16]. Among the armamentarium of available PROMs, the Edmonton Symptom Assessment System-Revised: Renal (ESASr: Renal) and Integrated Palliative Care Outcome Scale-Renal (IPOS-Renal) [17, 18] have the benefit of brevity, are available in several languages and include questions that a caregiver can complete on the patient's behalf. The latter is particularly important in older adults, who may experience barriers to PROM engagement because of cognitive impairment or being too unwell to complete the questionnaire.

The contextual nature of symptom experience (i.e. its dependence on psychosocial, situational and performance-related factors) and the high prevalence of geriatric impairments among older adults with ESKD means that the comprehensive geriatric assessment (CGA) may provide a suitable framework for symptom assessment and management in this population [11, 19]. The CGA describes a multidimensional and multidisciplinary method of assessing older adults’ medical, psychological, nutritional, functional, social and environmental needs, and is a well-evidenced intervention in acute medicine [20]. In a recent systematic review of 28 randomized control trials (conducted in the general population), the implementation of CGA was found to improve HRQoL and reduce caregiver burden [21]. However, further research on the effect of the CGA on symptom burden and HRQoL in the ESKD population is still required.

## TREATMENT DECISIONS: HOW AND WHEN

Nephrologists tend to focus on kidney disease-specific management rather than the broader issues affecting older people with advanced CKD. Comorbidity, frailty and psychosocial issues present a unique challenge to ESKD decision-making in this population. Table 2 outlines some recommendations from major society guidelines for managing older people with multi-morbidity, and are relevant to nephrology practice [22, 23]. A recent study of challenges in the shared-decision making (SDM) process found that nephrologists tend to prioritize dialysis planning, despite the patient's desire for greater emphasis on other personal and medical problems [24].

**Table 2:** Major guideline recommendations for managing older people with multi-morbidity

UK National Institute for Health and Care Excellence (2016) [22]
Management decisions should focus on:
• How health conditions and their treatments interact and how this affects quality of life
• The individual's needs, preferences for treatments, health priorities, lifestyle and goals
• Benefits and risks of following recommendations from guidance on single health conditions
• Improving quality of life by reducing treatment burden, adverse events and unplanned care
• Improving coordination of care across services
American Society of Geriatrics (2019) [23]
• Identify and communicate patients’ health priorities and trajectories
• Stop, start or continue care based on health priorities, potential benefit vs harm and burden, and health trajectory
• Align decisions and care among patients, caregivers and other clinicians with patients’ health priorities and health trajectory

In SDM, clinicians provide evidence-based and unbiased information on disease, prognosis and treatment options, while also seeking to elicit the person's beliefs, values and preferences. An overview of the information exchange that might occur as part of SDM in advanced CKD is detailed in Table 3. Although SDM is portrayed as a two-way process in which the person and clinician are equal partners, in reality, the discussion is led by the clinician who must give and elicit information. The power differential in the therapeutic relationship presents a further challenge to SDM in practice [24].

**Table 3:** Information exchange between person and clinician as part of SDM process

Person with advanced kidney disease	Nephrology healthcare team
**Lifestyle** • Functional status • Social activities • Employment • Travel **Social support** • Family/friends • Need for formal carers **Goals** (*‘What really matters to you?’*): • Quality of life • Maintaining independence • Survival (to a special event) • Travel	**Kidney disease prognosis** Anticipated rate of decline in kidney function to ESKD **Overall prognosis (considering comorbidity)** Realistic expectation of being alive in 1, 2 and 5 years, with and without dialysis. **SC** • Treatment decisions will always involve the patient • Interventions to reduce suffering and improve QoL, regardless of disease-specific treatments **Dialysis vs CKM** Realistic, evidence-based and unbiased information on dialysis (all modalities) and CKM What to expect (with and without dialysis) in terms of: • Physical function • Cognitive function • Symptom burden

QoL: quality of life.

Discussing kidney and overall prognosis may provide the person and caregivers a valuable insight into the seriousness of their condition and set the stage for advance care planning. Studies in the UK and USA have demonstrated a significant mismatch between the anticipated life expectancy reported by patients and their clinicians. Compared with healthcare professionals, people with advanced CKD generally tend to predict better survival outcomes, which has important implications for treatment expectations and care goals. Unsurprisingly, people who anticipated a shorter survival tend to favour care focused primarily on symptom relief, whereas those who predicted longer survival are more likely to opt for life-extending treatments [25–27].

Although prognostic tools for life expectancy and rate of kidney function decline are indicative, uncertainties remain. For example, the increasingly used Kidney Failure Risk Equation overestimates ESKD risk in older people because of the competing risk for death with advanced age [28]. Furthermore, survival prognostic scores dialysis relate to specific populations and may not accurately estimate survival for a given individual. Establishing physical function and the need for assistance in activities of daily living provides a reasonable indication of prognosis [29], quality of life and treatment burden on dialysis [30].

When counselling an older person on their treatment options, it is important not to overplay the benefits of dialysis. There is a natural tendency for both parties to approach dialysis initiation with the anticipation that it will lead to improvements in HRQoL. It can be helpful to include statements such as, ‘Dialysis is not going to make you young again’, and discuss the risk for functional and cognitive decline after dialysis initiation [9] and how this might impact caregiver burden [31].

The timing of treatment discussions also needs to be individualized. A standard approach is to start counselling the person on their options (i.e. dialysis or CKM) once the eGFR is 15–20 mL/min/1.73 m^2^. This approach improves the rate of vascular access formation but also has many downsides. Conversations held too early can have a negative psychological impact, and given that older people tend to experience slower kidney disease progression and a higher competing risk of death from other causes [32], the anxiety provoked through the contemplation of ESKD may not be justified. Over-zealous planning in this population can also result in the formation of vascular access which will never be used and psychological distress for the person [33], and represents unnecessary use of healthcare resources. A previous study of pre-emptive vascular access formation in older adults demonstrated that 15.1% died before the vascular access could be used and 17.5% survived dialysis free at the end of the 2-year follow-up [34]. Another approach is to discuss treatment options expectantly once it seems likely that the person will progress to ESKD. The principle disadvantage of this approach, however, is that there is a greater risk for hospitalization and ‘acute start’ haemodialysis (HD) via a central venous catheter. This denies the individual the opportunity to consider home dialysis or CKM.

## ADVANCE CARE PLANNING

Although several ACP interventions have been studied in the dialysis setting [35–38], research focused on the pre-dialysis advanced CKD population is scarce [39]. Among older adults approaching ESKD, ACP discussions may improve the person's understanding of their illness and the treatment options available to them (i.e. dialysis or CKM), identify care goals and facilitate end-of-life discussions [5, 6, 39]. Where the person has decided to undergo dialysis it is important that the ACP process addresses the issue of potential dialysis withdrawal in the future. Furthermore, ACP discussions provide a platform for the clinical team to explore perceptions of illness and treatment with the patient's caregivers, allowing an opportunity to prepare them for the role and responsibility of being a surrogate decision maker [14].

The SPIRIT (Sharing Patients’ Illness Representations to Increase Trust) method used by Song *et al*. is an example of a structured framework for ACP [40]. A care goals document is completed at the end of a dedicated ACP session, which should capture the person's preferences for treatment, escalation, resuscitation and end-of-life care. Ensuring that the person understands the implications of their decisions is an important part of ACP. After a period of 2 weeks, the decisions contained in the document are again reviewed with the patient and their caregivers, before being submitted to the person's health record. Recognizing that a person's health and preferences will change over time, ACP decisions should be reviewed at least annually, but also in the setting of a significant intercurrent event, clinical deterioration or at the patient's request [40].

Incorporating ACP into the care of older people with advanced CKD requires that healthcare professionals receive dedicated training. A further barrier to meaningful communication is the dearth of prognostic data which makes predicting outcomes with dialysis or CKM difficult on an individual basis. It might be more meaningful to explore a variety of best and worst case scenarios with the person (with and without dialysis) to elicit their care preferences [41, 42].

A recent analysis found that nephrologists did not view ACP as their responsibility, and tended only to discuss ACP when they were asked to do so by their patients. Interestingly, most of the patients in this study perceived ACP as an opportunity to discuss prognosis and treatment. Nephrologists viewed ACP as an exercise in documenting decisions on resuscitation and treatment limitations, rather than a meaningful dialogue in which to explore the person's care preferences [43].

## CONSERVATIVE KIDNEY MANAGEMENT AND SUPPORTIVE DIALYSIS IN THE OLDER ADULT

### Conservative kidney management

CKM is a non-dialysis treatment strategy for advanced CKD that is considered a suitable alternative to dialysis among certain older adults. Emerging evidence suggests that CKM is associated with comparable survival and HRQoL outcomes to dialysis in adults aged over 80 years with significant comorbidity [4–7].

What constitutes CKM will vary between individuals, and is largely dependent on the person's care goals, functional status and disease state. A comprehensive framework for CKM is outlined by the Alberta Supportive Kidney Care group [44].

In the absence of uniform guidance for CKM, clinicians need to develop an understanding of an individual's care goals early on to guide management and tailor interventions to meet these specific needs. Table 4 outlines guideline-based recommendations for managing advanced CKD progression and the metabolic complications encountered in people following a conservative pathway [45]. These interventions are not prescriptive and should be adjusted to meet the care goals of the individual. As with any ESKD treatment, the appropriateness of specific interventions should be evaluated periodically.

**Table 4:** Specific kidney disease management considerations in CKM for the older adult

Complication	Recommendation	Rationale and goals
Hypertension	*Aim for individualized BP targets* • Treat very high BP, avoid very low diastolic BP • Arbitrary target range: <150 mmHg systolic and >70 mmHg diastolic BP • Choice of BP*-*lowering agents depends on comorbidities • Use loop diuretics to treat hypertension when there is evidence of fluid overload • Consider discontinuation of RAAS inhibitors	• Minimize the risk of falls and syncope by avoiding excessively tight BP control. Hypotension (diastolic BP <60 mmHg) is associated with increased mortality in older adults • Preserve functional and cognitive faculties • Slow CKD progression
Fluid overload	*Aim to prevent fluid overload* • Dietary sodium restriction ≤6 g/day • Loop diuretics if necessary	• Decrease symptom burden related to fluid overload (i.e. dyspnoea, dependent oedema) • Consider the balance between reducing risk of fluid overload (by dietary restriction and diuretics) and the impact these interventions have on nutritional well-being and QoL
Anaemia	*Aim to prevent symptomatic anaemia* • Consider iron supplementation in functional and absolute iron deficiency, targeting a ferritin 200–500 mg/L and transferrin saturation 20%–30% • Consider treatment with an ESA for patients with Hb <10 g/dL • If Hb >12 g/dL, stop ESA or decrease dose	• Treatment should be provided to address symptomatic anaemia (i.e. fatigue, dyspnoea) rather than specific laboratory targets • Interventions may be of limited benefit with poor functional reserve (i.e. bedbound) or at the end-of-life
Disorders of calcium and phosphate balance	*Aim to prevent symptoms and maintain adequate nutritional intake* • Consider dietary phosphate restriction and/or phosphate binders (depending on the patient's preference and tolerance) • Consider activated vitamin D supplementation • Routine monitoring of PTH and the introduction of pharmacological agents to meet specific PTH targets is not recommended in older adults on a conservative pathway	• Treatment is focused on symptom relief (i.e. may help symptoms of restless legs, myalgia, pruritus and pseudogout) • In patients with eGFR <60 mL/min/1.73 m^2^ hyperphosphatemia is associated with increased mortality. It is unclear if this also accounts for patients with limited life expectancy
Hyperkalemia	Aim to maintain serum potassium <5.5 mmol/L • Dietary potassium restriction and/or potassium binders (depending on the patient preference and tolerance) • Consider discontinuation of RAAS inhibitors • Address metabolic acidosis	• Reduce the risk of cardiac dysrhythmia • Consider balance between tight dietary and pharmacological potassium control and its impact on nutritional status and QoL
Metabolic acidosis	*Aim to maintain serum bicarbonate ≥20 mmol/L but <26 mmol/L* • Consider oral sodium bicarbonate (if tolerated)	• Might have positive impact on fatigue, sarcopenia and bone health • Theoretically slows progression of CKD
Dyslipidemia	*Consider benefit of ongoing treatment with lipid-lowering agents*	• Improvement of QoL without negative effect on cardiovascular morbidity in last years of life

QoL: quality of life; BP: blood pressure; ESA: erythropoietin-stimulating agents; RAAS: renin–angiotensin–aldosterone system; Hb: haemoglobin; PTH: parathyroid hormone.

**Table 5:** Comparison of PD and HD in the older adult

PD	HD
Advantages	
• Home-based treatment: reduces risk of nosocomial infection, avoids burden associated with transport, is more flexible than in-centre HD (days off treatment possible if sufficient residual kidney function)	• Dialysis procedure performed by others
• Assistance is possible if required, either by family/friend or trained professional	• In-centre HD represents an important social outlet for many older people
• Greater preservation of residual kidney function	• Pathways for establishing a person on HD are well developed within most nephrology units
• Less haemodynamic disturbance and not associated with same ‘washed out’ feeling reported by patients undergoing HD	
• Does not require routine anticoagulation	
Disadvantages	
• Impact on quality of life (frequency of exchanges on CAPD, adjusting to nocturnal treatment with APD)•	• Inferior vascular access outcomes with increasing frailty; frailer patients typically require a tunnelled CVC which is associated with an increased risk for infection•
• Where assistance is not possible, access to PD is reduced among older and frail adults	• Impact on quality of life (need to factor in transport time and ‘washed-out’ feeling experienced by many people after a standard HD session
• Risk of peritonitis	• Risk of intradialytic hypotension and more rapid loss of residual kidney function compared with PD
• Clinical teams tend not to be as comfortable dealing with PD-related issues	

CAPD: continuous ambulatory peritoneal dialysis; APD: ambulatory peritoneal dialysis; CVC: central venous catheter.

**Table 6:** Training in SC

Essential skills in a SC curriculum
• Comprehensive assessment of the frail older adult (understanding components of comprehensive geriatric assessment)
• Ability to provide a prognostic assessment
• Ability to deliver clear information on dialysis and non-dialysis approaches to ESKD management
• Knowledge of relevant SC clinical guidelines
• Develop advanced communication skills with ability to perform advance care planning and to address topics such bad news, limited prognosis and uncertainty
• Clear understanding and approach to the process of dialysis withdrawal
Mode of teaching and learning
• Didactic fact-based teaching
• Simulated communications skills workshops
• Regular attendance at supportive care clinics (in both non-dialysis and dialysis settings)
• Assessment of trainee by a specialist SC practitioner (direct observation of the trainee conducting a clinical interaction)

### Supportive dialysis: adapting treatment to meet the needs of older people

Older adults with ESKD are more likely to prioritize HRQoL outcomes over survival [10], so achieving specific biochemical and small solute removal targets may not confer a meaningful benefit in this population [46, 47].

A slower decline in eGFR and residual kidney function (RKF) should also enable a reduction in the prescribed dose for both peritoneal dialysis (PD) and HD, which may improve the person's perception of treatment burden and fits with existing multi-morbidity guidelines [22, 23]. Table 5 provides an overview of considerations in each dialysis modality for the older person with ESKD.

### Peritoneal dialysis

PD can be used successfully in older people with ESKD [48] and results in similar HRQoL outcomes to in-centre HD. Among those able to perform autonomous PD, reported illness intrusiveness is lower than with in-centre HD [49]. Furthermore, assisted PD (asPD), whereby the PD technique is performed by a formal or informal caregiver, is associated with better treatment satisfaction among older people compared with HD [50]. Healthcare worker–delivered asPD increases the access that older people have to home dialysis therapies, but formal asPD programmes remain unfunded in many healthcare systems [51, 52].

One of the authors (E.A.B.) has developed a programme of supportive two-exchange assisted continuous ambulatory PD with embedded ACP which appears to provide control of uraemic symptoms in the older person who cannot do autonomous PD or in-centre HD [53].

Although poor functional status is often perceived as a contraindication to PD, evidence suggests that functional impairments are a predictor of poor HRQoL and treatment burden for both PD and HD, regardless of a person's age [30].

### Haemodialysis

Conventional thrice-a-week HD in older patients is associated with a significant treatment burden and loss of independence. The DOPPS study found that 10% of adults undergoing maintenance HD required a 12-h recovery following a standard HD session; this became more common with increasing age and comorbidity [54], which leads to a significant loss of quality time.

In light of evidence that frailty portends poor vascular access outcomes and the need for more frequent interventions to maintain patency, the KDOQI (Kidney Disease Outcomes Quality Initiative) guideline for vascular access now recommends a ‘patient first’ approach to such decisions [55], and tunnelled venous catheters may represent a reasonable first-line option in older individuals living with frailty.

Preservation of RKF is associated with improved outcomes among people on dialysis [56] and may be preserved in HD by adopting an incremental approach (shorter or less frequent dialysis sessions initially). The elderly are particularly prone to episodes of intra-dialytic hypotension [57], which can have a detrimental effect on RKF. This provides further impetus to consider incremental HD regimens, which factor RKF into clearance estimations, to improve mortality outcomes and quality of life. However, patients on incremental regimens require careful monitoring so that the dialysis dose can be increased as RKF declines.

Nonetheless, a recent large observational study found no difference in survival between people treated with incremental HD versus standard HD. However, lower RKF was associated with worse mortality outcomes [58]. A recent systematic review suggests that incremental HD can successfully allow deferment of thrice-a-week dialysis for about 1 year without increasing mortality risk [59].

## SC IN THE FAILING KIDNEY TRANSPLANT

Ageing and increased access to transplantation among older adults [60] means that SC is equally relevant in the setting of kidney transplant. SC strategies are applicable in the failing transplant, but also among people with a functioning transplant who might be approaching the end-of-life for another reason. The latter represents an important consideration given that malignancy is one of the main causes of death in this population [61].

There is a dearth of literature examining SC in kidney transplant, but, extrapolating from other solid organ transplant populations [62–64], it is likely that SC and palliative medicine are under-utilized. A study of North American patients showed that any engagement with transplantation (i.e. previous or current transplant listing, functioning transplant or failed transplant) increases the likelihood of life-extending interventions (i.e. resuscitation, ventilation and artificial nutrition) in the last 30 days of life [65]. Furthermore, those who have engaged with transplant are less likely to be enrolled in hospice care or discontinue dialysis. It is possible that engagement with transplantation and its association with ‘cure’ and prolongation of life colours prognostic perceptions and may undermine SC processes such as ACP, despite evidence that such patients wish to receive SC interventions [66, 67].

## DIALYSIS WITHDRAWAL

Once a person feels that the burden of dialysis far exceeds the benefit, they may choose to withdraw from treatment [68]. Seeking to address any reversible factors, such as symptom burden or depression, should always precede compassionate support for withdrawal. On some HD programmes, dialysis withdrawal accounts for over 30% of deaths [69], reflecting, perhaps, the ageing dialysis population and success of transplant programmes in younger adults.

As mentioned earlier, dialysis withdrawal discussions should form part of the ACP process and are an essential component of care in the older and multi-morbid adult with advanced CKD. Although this represents a difficult issue for both patients and clinicians, anticipating dialysis withdrawal early on should ultimately make the decision and process less challenging for the patient and clinical team should the need arise. The British Geriatric Society has published useful guidance on ACP, which includes suggestions on how difficult conversations might be approached in practice [70]. The language used in these discussions is important, and care should be taken not to use terms which may have negative connotations such as ‘stopping dialysis’.

The mean survival post-dialysis withdrawal is 10 days, but patients should be sensitively counselled that in the presence of RKF they may live longer. One study reported post withdrawal survival up to 48 days [71]. Factors associated with an increased likelihood for dialysis withdrawal include female sex, Caucasian ethnicity, old age, comorbidity and HD (rather than PD) [72, 73]. One Dutch study found that 26% of patients withdraw within the first year of dialysis [71], suggesting a need for improved education on treatment choices in the pre-dialysis period.

## SC ACROSS CULTURES

The principles of candour and patient autonomy are embedded in the framework of Anglo–American medical ethics. In many parts of the world, however, the cultural norm is protecting the patient from the truth of their condition, decision making by the family, and a tradition of familial piety, where it is considered dishonourable not to do as much as possible for an unwell parent [74]. These cultural differences lead to conflict between patients, families and clinicians. Therefore, strategies to reduce cross-cultural miscommunication are needed [75].

Cross-cultural communication should acknowledge individual cultural traditions, avoid generalizing a patient's beliefs or values, and consider one's own beliefs, values and experiences [76]. After the clinician has acquired an understanding of the type of information desired, how the information is conveyed is equally important. Patients and families may have language barriers and low health literacy, further complicating their ability to process and act on critical medical information [77]. A recent analysis comparing survival among ethnic minorities (Black, Asian and Hispanic) and whites found that dialysis discontinuation was less common among those belonging to an ethnic minority following hospitalization with stroke, lung cancer, dementia and ‘failure to thrive’. The authors suggest that family support, religious belief, education on treatment options and trust in clinicians may account for the observed differences in voluntary dialysis withdrawal [72]. A randomized control trial among a predominantly Black American HD cohort found that ACP was associated with less depression and post-traumatic distress in the patient's surrogate after death [37].

## ESTABLISHING THE FRAMEWORK AND INFRASTRUCTURE FOR AN SC PROGRAMME

**Figure 2:**
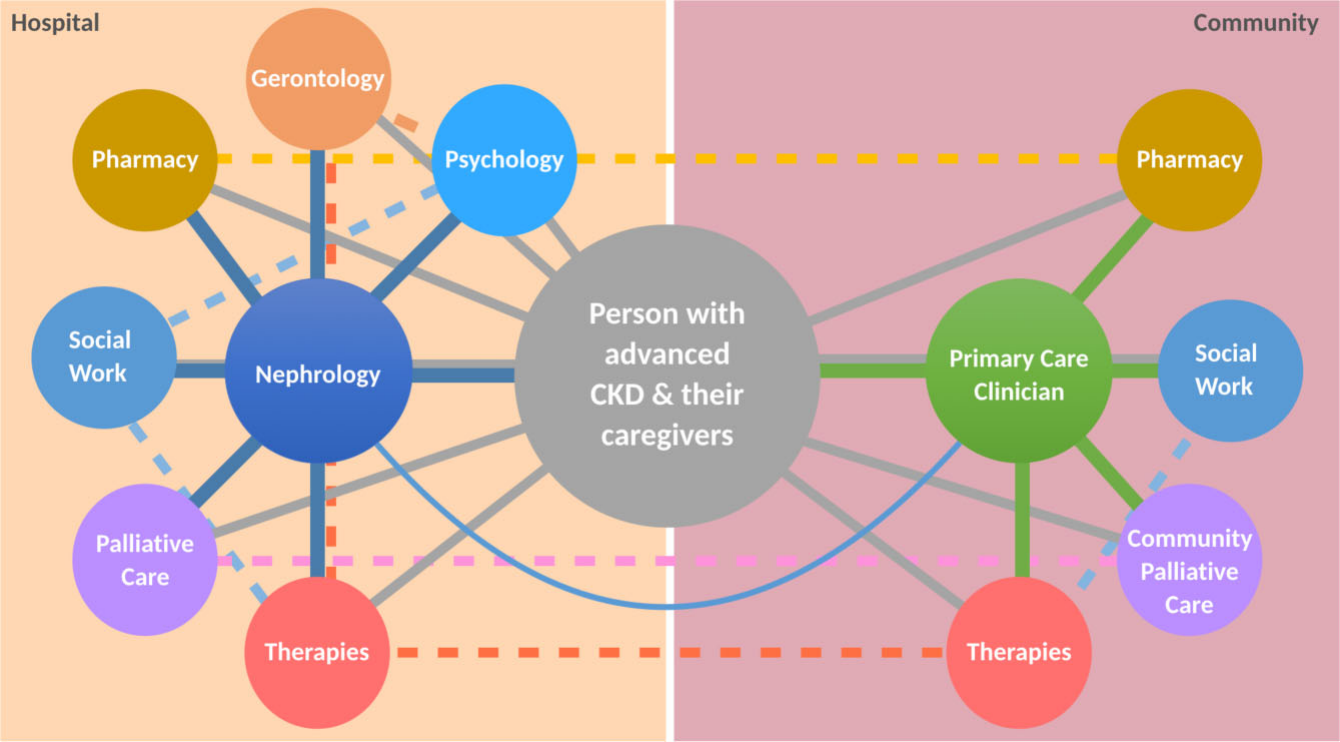
Networks in SC.

The complex nature of SC means that care networks are vital, but developing them requires time and effort. Stakeholders in the SC network for some units might include nephrologists, geriatricians, frailty specialist nurses, therapies, counselling services, pharmacy, primary care, hospice and community palliative teams, but this will vary depending on available resources (Fig. 2). A lead clinician (or SC champion) with the ability to influence change and leverage resources is crucial to integrating SC into routine nephrology care. Trained and dedicated SC staff are key, but it is equally important that all nephrology clinicians have basic SC experience and can recognize when it might be appropriate to refer a person for sub-specialist evaluation.

Patient involvement in the design of SC programmes ensures that they are relevant and accessible to the service users [78]. Patients can also assist in the development of educational materials [79], which should sensitively describe prognosis and treatment choices (ideally in multiple formats and languages).

For clinicians, good clinical guidelines in SC (covering pharmacological therapies and permissive treatment targets) are essential [45, 79] to reduce unnecessary intervention. In constructing these guidelines, nephrologists should place a particular emphasis on interventions that improve quality of life and limit treatment burden, acknowledging the short life expectancy that most older people with advanced CKD experience.

## TRAINING CLINICIANS IN SC

The KDIGO Controversies in Supportive Care Conference in 2015 [14] highlighted SC training for nephrology clinicians as a priority. A previous qualitative study of US and UK nephrologists identified inadequate training as a perceived barrier to adopting SC practices [80]. More recently, a survey of 250 mixed healthcare professionals in South London demonstrated that only 50% of nurses felt confident discussing SC strategies with multi-morbid patients, and only 12% felt they had the pre-requisite knowledge to discuss prognosis. Amongst the physicians surveyed, 80% reported having found an ACP created by someone else helpful, while fewer than 30% had completed one themselves [81].

A SC programme should provide evidence-based instruction on performing prognostic assessments, managing symptoms [80], and using SDM and ACP in practice (see Table 6). Nephrology trainees may benefit from training secondments in geriatric and palliative medicine, specialties that place a particular emphasis on SDM and working within multi-disciplinary teams.

Examples of SC programmes for nephrology trainees include NephroTalk and REnal specific Advanced Communication Training (REACT). The NephroTalk programme was developed and piloted among US trainees and improved knowledge of fundamental CKM concepts. Following completion of the hybrid course, trainees reported increased confidence and proficiency in delivering bad news [82]. The REACT programme was developed in the UK through a multi-professional and patient engagement process. It was piloted among nurses, healthcare assistants and physicians at two National Health Service hospitals and led to increased confidence among staff in conducting end-of-life discussions [83]. Both programmes emphasize that attending to emotional cues and ensuring strong emotions are acknowledged can allow factual information to be absorbed, which might otherwise be lost to the patient.

Culture change and SC training for nephrologists may also help stem burn-out and moral distress, an issue that was highlighted by a recent study of US nephrology fellows. This study found high levels of moral distress among nephrology trainees relating to situations where patients were undergoing dialysis despite perceived futility or terminal illness [84].

## CONCLUSION

Tailoring care to meet the needs of older individuals with advanced CKD is challenging and requires considerable time, thought and effort on the part of nephrology teams. Integrating SC principles (i.e. shared-decision making, advance care planning and symptom control) into nephrology practice should help ensure that interventions remain aligned with the person's care goals and improve quality of life. The underlying aim of SC is to reduce suffering, and this should be viewed as a therapeutic priority for all clinicians involved in the care of older adults with advanced CKD, regardless of the person's decision to follow a dialysis or non-dialysis treatment pathway.

SC in nephrology is an emerging discipline, and determining its true value within advanced CKD care requires further research [85]. Epidemiological and outcome data would permit the development of a clear definition for SC and evidence-based guidelines, and provide the impetus for greater investment in the area. Perhaps more important is culture change and SC training for nephrologists. The limited opportunities for SC training represents a significant unmet need in nephrology, which requires urgent attention if our speciality is to adequately address the care needs of an older and frailer cohort of people with advanced CKD going forward.

## Data Availability

No new data were generated or analysed in support of this research.
